# The Number of Pulses Needed to Measure Corticospinal Excitability by Navigated Transcranial Magnetic Stimulation: Eyes Open vs. Close Condition

**DOI:** 10.3389/fnhum.2017.00121

**Published:** 2017-03-21

**Authors:** Shahid Bashir, Woo-Kyoung Yoo, Hyoung Seop Kim, Hyun Sun Lim, Alexander Rotenberg, Abdullah Abu Jamea

**Affiliations:** ^1^Department of Physiology, Faculty of Medicine, King Saud UniversityRiyadh, Saudi Arabia; ^2^Berenson-Allen Center for Noninvasive Brain Stimulation, Beth Israel Deaconess Medical Center, Harvard Medical SchoolBoston, MA, USA; ^3^Department of Physical Medicine and Rehabilitation Medicine, Hallym University Sacred Heart HospitalSeoul, South Korea; ^4^Hallym Institute for Translational Genomics and Bioinformatics, Hallym University College of MedicineSeoul, South Korea; ^5^Department of Physical Medicine and Rehabilitation, National Health Insurance Ilsan HospitalSeoul, South Korea; ^6^Neuromodulation Program, Division of Epilepsy and Clinical Neurophysiology, Department of Neurology and the F.M. Kirby Neurobiology Center, Boston Children’s HospitalBoston, MA, USA; ^7^Department of Radiology and Medical Imaging, College of Medicine, King Saud UniversityRiyadh, Saudi Arabia

**Keywords:** motor evoked potential, electromyography, muscles, transcranial magnetic stimulation

## Abstract

**Objective**: Motor evoked potentials (MEPs) obtained by transcranial magnetic stimulation (TMS) enable measures of the corticospinal excitability (CSE). However the reliability of TMS-derived CSE measures is suboptimal due to appreciable pulse-to-pulse MEP amplitude variability. We thus calculated how many TMS–derived MEPs will be needed to obtain a reliable CSE measure in awake adult subjects, in the eyes open (EO) and eyes closed (EC) conditions.

**Methods**: Twenty healthy adults (70% male) received 40 consecutive navigated TMS pulses (120% resting motor threshold, RMT) in the EO or EC conditions on two separate days in randomized order.

**Results**: For either the EO or EC condition, the probability that the 95% confidence interval (CI) derived from consecutive MEP amplitude measured included the true CSE, increased when the number of consecutive stimuli increased (EO: *p* = 0.05; EC: *p* = 0.001). No significant effect of RMT, Mini-Mental State Examination (MMSE) score, or gender on the CSE estimates was identified. At least 34 consecutive stimuli were required to obtain a most reliable CSE estimate in the EO condition and 31 in the EC condition.

**Conclusion**: Our findings indicate that >30 consecutive MEPs may be necessary in order to obtain a CSE measure in healthy adults.

## Introduction

Transcranial magnetic stimulation (TMS) is a non-invasive, painless and safe method for focal cortical stimulation induced by generating high-intensity magnetic field by passing a brief intracranial electrical current through a magnetic coil (Barker et al., 1985; Kobayashi and Pascual-Leone, 2003). When applied to the primary motor cortex (M1) and coupled with hand surface electromyography (EMG), TMS is often used to measure corticospinal excitability (CSE) as reflected in the amplitude or area under the curve of a hand muscle TMS-induced motor evoked potential (MEP). CSE is generally equated to the average MEP amplitude, resulting from multiple stimuli to the same region at identical intensities (Kiers et al., 1993; Magistris et al., 1998; Rösler et al., 2002; Pitcher et al., 2003; Darling et al., 2006; Mars et al., 2007; Bashir et al., 2011, 2014; Roy Choudhury et al., 2011; Lewis et al., 2014; Cakar et al., 2016). Yet MEP amplitudes vary considerably from pulse to pulse in individual subjects (Magistris et al., 1998; Rösler et al., 2002; Pitcher et al., 2003; Roy Choudhury et al., 2011), and this leads to compromising reliability of CSE metrics. In most protocols, 6–10 MEPs are averaged for the CSE estimate (Magistris et al., 1998; Rösler et al., 2002; Pitcher et al., 2003; Bashir et al., 2011, 2014; Roy Choudhury et al., 2011; Lewis et al., 2014). Yet number of TMS pulses and MEPs that are necessary to obtain a reliable CSE estimate is unknown? We therefore stimulated the motor cortex in healthy volunteers, and measured the number of intrinsic hand muscle MEP amplitudes that would be needed to obtain a reliable CSE estimate.

Since the instantaneous state of cortical motor neuron and cortical interneuron activity likely plays a role in MEP amplitude variability in TMS experiments, we obtained these measures in two conditions: awake with eyes open (EO) and eyes closed (EC), which previously have been demonstrated to modulate cortical and/or spinal motoneural output (Rossini et al., 1991).

In the present experiment, we hypothesized to test whether the number of pulses needed to establish a CSE metric from the first dorsal interosseous (FDI) muscle representation is mediated by factor for which studies may not reliably control: activation of the visual system as defined by the EO and EC condition.

## Materials and Methods

### Subjects

Twenty right-handed control subjects (age 19–31 years; 70% males) participated in this study (Table 1). The Edinburgh Handedness Inventory (EHI; Oldfield, 1971) was administered to verify that all subjects were right-handed (right-handedness 1.93 ± 0.27). The participants had normal cognitive scores (normal range: 28–30) as indexed by Mini-Mental State Examination (MMSE; Folstein et al., 2001).

**Table 1:** **Demographic table**.

Age (years)	23.9 ± 2.93
Gender	14M, 6F
Mini-mental state examination	30
Weight (Kg)	83 ± 6.53
Resting motor threshold	48.5% ± 5.80% of maximum stimulator output
Motor evoked potentials (Eye closed)	858 ± 520 μV
Motor evoked potentials (Eye open)	1041 ± 497 μV

Participants underwent two experimental sessions separated by 1 week using a within-subjects, a crossover randomized design, during which they underwent M1 TMS with MEP monitoring in either the EO or EC condition.

Participants had no contraindications to receive TMS and were not taking any medication known to affect motor cortical excitability at the time of the study (Rossi et al., 2009). Furthermore, neurological examination of the subjects revealed no abnormal signs to suggest any underlying neurological or psychological condition. The investigation was carried out in accordance with the latest version of the Declaration of Helsinki and was approved by the local Review Board (King Khalid University Hospital). All participants gave their written informed consent prior to enrollment in the study.

### Experimental Set-Up

The stimulation setup consisted of a frameless stereotaxic system for navigation (Visor2 ANT; Netherlands) connected to TMS system (Mag Pro X100 (MagVenture, Denmark)). All subjects had a high-resolution anatomical head magnetic resonance image (MRI) that was used to ensure stimulation accuracy during and across sessions. In each follow-up session co-registration errors to the MRI’s surface landmarks were matched to ≤3 mm.

Surface EMG signals were recorded from FDI muscle in which active electrodes were attached and reference electrodes were placed over the metacarpophalangeal joints. These EMG signals were band-pass filtered (8−500 Hz), amplified, displayed and stored for off-line analysis. The TMS system delivered trigger pulses that synchronized with EMG systems.

During the sessions participants sat on a comfortable chair with their hands in a supine position on their laps, wearing earplugs, and remaining silent to avoid speech-induced modulation of cortical (Bashir et al., 2011, 2014).

### TMS Protocol

All subjects had undergone a high-resolution T1-weighted structural MRI scan before TMS session. Imaging data were fed to the navigation software (Visor2, ANT, Netherlands) for automatic 3D brain reconstruction that was used to guide navigation and deliver TMS over M1 (“hot spot”). In each TMS session, the motor cortical output was mapped carefully for the optimal representation of the FDI muscle on left hemisphere (dominant hemisphere) as previously described (Bashir et al., 2011, 2014).

The TMS coil was held tangentially to the skull with the coil handle pointed 45° posterior-laterally to the sagittal plane, which the coil and the induced current on the cortex orientated perpendicularly to the anatomically defined central sulcus and induces a posterior-to-anterior current direction (Bashir et al., 2011, 2014).

Each individual’s resting motor threshold (RMT) was determined as the minimum %maximal stimulation output (MSO) TMS intensity that produced at least 5 out of 10 MEPs ≥50 μV, that was obtained at that location. The site which evoked MEPs with highest amplitude (henceforth, the hot spot) was then marked on each subject’s MRI. Nearly always, the hotspot did not change; sometimes, the coil position needed a minor adjustment in roll or pitch. Forty single stimuli 4–8 s apart were delivered to the hot spot at an intensity of 120% of RMT to determine baseline MEP peak to peak amplitude for the EO and EC conditions.

### MEP Data Analysis

To minimize the variability of TMS-induced individual MEPs, they were screened for artifact and for signal indicating voluntary contraction, and excluded (<1%) if the root mean square EMG exceeded 5 mV during the 50-ms period immediately preceding the onset of the TMS pulse as previously described (Cuypers et al., 2014). All subjects data were analyzed for 2 blocks (EO and EC) of 40 consecutive TMS stimuli at 120% RMT. The order of these two conditions was randomized and counterbalanced across participants. For each subject, the average MEPs of EO or EC was calculated for subsets of consecutive stimuli:

$$\bar{\text{MEPn}}\;=\;\frac{1}{n}\sum_{1}^{n}MEP,\text{ where}\;n\text{ ​}:\text{2}\ldots \text{40}.$$

The generalized estimating equation (GEE; SAS Institute, Cary, NC, USA) was used to determine the contribution of the EO and EC condition, as well as gender and MMSE score on the $\bar{\text{MEPn}}$. To evaluate the accuracy of the MEP estimates, a 95% confidence interval (CI) was calculated using all 40 stimuli for each subject, per condition as previously described (Cuypers et al., 2014). Based on both the MEPn values and the CI, it is possible to determine whether MEPn is included in CI, yielding a binary variable (0 = not included in the CI, 1 = included in the CI) as previously described by Cuypers et al. (2014). The number of consecutive stimuli required as a function of the probability of hitting the 95% CI.

## Results

**Figure 1:**
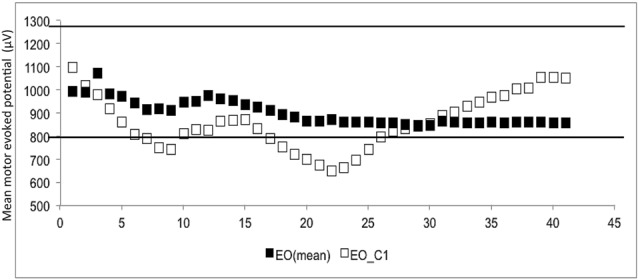
**The mean and 1st subject data of eye open (▪ = mean, □ = the data of 1st subject).** The *Y*-axis shows the motor evoked potential (MEP) amplitude (μV), while the number of transcranial magnetic stimulation (TMS) stimuli (*n*) is shown on the *X*-axis. White dots represent the individual (raw) MEPs, whereas the black dots represent the average of consecutive MEPs (MEPn). Dashed lines represent the 95% confidence interval (CI), which is based upon all 40 stimuli.

The CSE estimates did not differ between the EO and EC conditions. The probability that MEPn fell within the CI_40_ CSE estimate increased with successive TMS pulses (Figure 1). The GEE analysis showed that the estimate of the CI increased when the number of consecutive stimuli increased (*p* = 0.05 for EO and *p* = 0.001 for EC).

**Figure 2:**
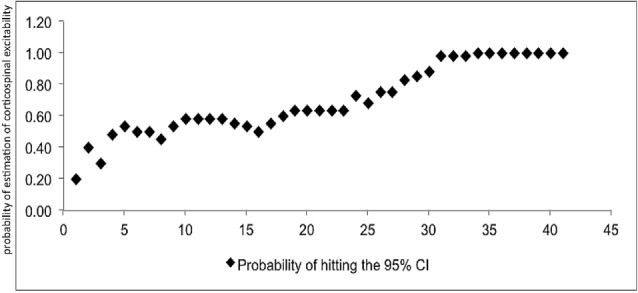
**Mean probability in 95% CI.** The *Y*-axis shows probability of inclusion in the 95% confidence interval (CI), while the number of transcranial magnetic stimulation (TMS) stimuli (*n*) is shown on the *X*-axis.

At least 31 consecutive stimuli were required in EO and 34 in EC condition to reach a 100% probability that the average of MEP fell within the 95% CI_40_ (Figure 2, Tables 2, 3). The GEE analysis showed a not significant effect of RMT, MMSE and gender on CSE estimates (all, *p* < 0.05). The interaction effect of RMT was not significant. But the age was significant with EC condition. As the age increased, the probability of 95% CI also increased. Female with EO: 29th, with EC: 24th and the male with EO: 31th, with EC condition: 34th (Table 4).

**Table 2:** **Probability table**.

Iteration	Probability of hitting the 95% CI
1	0.20
2	0.40
3	0.30
4	0.48
5	0.53
6	0.50
7	0.50
8	0.45
9	0.53
10	0.58
11	0.58
12	0.58
13	0.58
14	0.55
15	0.53
16	0.50
17	0.55
18	0.60
19	0.63
20	0.63
21	0.63
22	0.63
23	0.63
24	0.73
25	0.68
26	0.75
27	0.75
28	0.83
29	0.85
30	0.88
31	0.98
32	0.98
33	0.98
34–40	1.00

*The number of consecutive stimuli required as a function of the probability of hitting the 95% confidence interval (CI)*.

**Table 3:** **Probability table for eyes open (EO) condition**.

Iteration	Probability of hitting the 95% CI
1	0.20
2	0.40
3	0.25
4	0.40
5	0.45
6	0.45
7	0.40
8	0.35
9	0.45
10	0.50
11	0.45
12	0.40
13	0.45
14	0.45
15	0.45
16	0.40
17	0.45
18	0.55
19	0.55
20	0.55
21	0.55
23	0.60
22	0.55
24	0.65
25	0.60
26	0.70
27	0.70
28	0.85
29	0.90
30	0.95
31–40	1.00

*The number of consecutive stimuli required as a function of the probability of hitting the 95% confidence interval (CI) for EO*.

**Table 4:** **Probability table for eyes close (EC) condition**.

Iteration	Probability of hitting the 95% CI
1	0.20
2	0.40
3	0.35
4	0.55
5	0.60
6	0.55
7	0.60
8	0.55
9	0.60
10	0.65
11	0.70
12	0.75
13	0.70
14	0.65
15	0.60
16	0.60
17	0.65
18	0.65
19	0.70
20	0.70
21	0.70
22	0.70
23	0.65
24	0.80
25	0.75
26	0.80
27	0.80
28	0.80
29	0.80
30	0.80
31	0.95
32	0.95
33	0.95
34–40	1.00

*The number of consecutive stimuli required as a function of the probability of hitting the 95% confidence interval (CI) for EC*.

## Discussion

We demonstrate that >30 stimuli are required to obtain an accurate CSE estimate when MEP measures are obtained from the FDI muscle in healthy adults. Notably, this measure is independent of RMT, and gender. This basic practical information might play a crucial role for obtaining a reliable CSE in the TMS experiments. This study showed the intra-rater reliability, as every part of the data were collected by the same rater.

The interpretation of our results about CSE estimates by number of consecutive stimuli might be required different for other age groups and in disease populations, because our data was obtained in healthy young subjects. Second, all subjects were informed with respect to the experimental procedures due to uncertainty about their first TMS experience; which likely caused changes in attention, and this variable may differ from study to study. Furthermore, experimental set-up (navigated TMS, different coil types and shapes), EMG hardware and signal analysis for noise elimination can affect variability and reliability of the CSE measurements. Last our measures were obtained from the CI muscle (FDI) and a different numerical result may be seen in other muscle groups.

It is paramount that any reliability studies, such as ours, clearly outline the extent to which its results can be generalized. We thus proposed to extrapolate from our results with qualification. Reliability and reproducibility of protocol depends on the sample, the paradigm, the TMS setup and the operator (Beckerman et al., 2001).

The variability related to the TMS method should be similar across the laboratories, when one considers that we used stimulator and recording devices that is commonly available. This is the first TMS reliability study showing spatial stimulation stability within and between sessions using neuronavigation (Schönfeldt-Lecuona et al., 2005). It is noteworthy to emphasize that the lack of controlling in pitch and roll of TMS coil just by using marked scalps or swimming caps can cause measurement error as it allows spatial drift (Julkunen et al., 2009) and reduces the constancy and strength of intracranial electrical currents conveyed to a target (Cincotta et al., 2010).

The design of TMS experiments can be improved by the present study outcome.

## Author Contributions

All authors listed, have made substantial, direct and intellectual contribution to the work, and approved it for publication.

## Conflict of Interest Statement

The authors declare that the research was conducted in the absence of any commercial or financial relationships that could be construed as a potential conflict of interest.
